# A systemic vulnerability in child protection: the interprofessional gap in abuse and neglect recognition rooted in university curricula

**DOI:** 10.3389/fpubh.2026.1760670

**Published:** 2026-02-18

**Authors:** Merve Şahin, Osman Yılmaz Kartal

**Affiliations:** 1 Department of Child Development, Bayramiç Vocational School, Canakkale Onsekiz Mart University, Çanakkale, Türkiye; 2 Department of Educational Sciences, Faculty of Education, Canakkale Onsekiz Mart University, Çanakkale, Türkiye

**Keywords:** child abuse and neglect, interprofessional education, midwives, pre-school teachers, sustainable development goals

## Abstract

**Background/objectives:**

The integrity of child protection systems, a precondition for social sustainability, is compromised by an ‘interprofessional awareness gap’ among key sentinel professions, particularly within health and education. This study aims to empirically define this gap by comparing the preparedness of two critical future professional cohorts: final-year midwifery and pre-school education students. The objective is to reveal how their distinct university curricula shape foundational awareness of child abuse and neglect (CAN) before they enter professional practice.

**Methods:**

Using a causal-comparative design, this study assessed final-year midwifery (*n* = 246) and pre-school education (*n* = 115) undergraduates in Türkiye. Two validated psychometric instruments measured awareness across distinct subtypes of abuse and neglect, revealing the multifaceted nature of their understanding.

**Results:**

Findings revealed a significant disparity, with future midwives being less prepared to identify CAN than their education counterparts. This awareness deficit was most pronounced for subtle forms of maltreatment, such as emotional and economic abuse, and all dimensions of neglect. A student’s academic department was the sole significant predictor of awareness, outweighing the influence of academic progression or prior training.

**Conclusion:**

This gap represents a systemic vulnerability cultivated by siloed higher education paradigms. Midwifery’s traditional biomedical curriculum appears insufficient for imparting the holistic, socio-ecological perspective required for effective safeguarding. A paradigm shift toward mandatory Interprofessional Education (IPE) is imperative. Such reform is crucial for forging a shared professional ethos and a common language of risk, thereby creating a truly resilient and sustainable child protection framework.

## 1 Introduction

Originally an environmental science concept, sustainability has evolved to encompass a holistic vision for human survival. Social sustainability is at the core of this vision, resting on three pillars: preserving human capital, building fair societies, and protecting intergenerational well-being (1). This perspective aligns with the Adverse Childhood Experiences (ACEs) framework, which shows that child maltreatment forms a causal pathway to negative health outcomes throughout life (2). Protecting children is therefore not merely a matter of rights or morality, but a foundational requirement for a sustainable future. Indeed, child well-being is globally recognized as the starting point for lifelong health and human capital, making it a cornerstone of sustainable development (3). Consequently, any community that neglects the safety and health of its children undermines its own capacity for long-term resilience and prosperity (4).

This understanding is formally recognized in the United Nations’ 2030 Agenda for Sustainable Development. The Agenda frames good health not as a final goal, but as a driving force for progress in areas like poverty alleviation and quality education (5). While this principle is central to Sustainable Development Goal 3 (Good Health and Well-being), it is also deeply connected to Quality Education (SDG 4) and, most explicitly, to Peace, Justice, and Strong Institutions (SDG 16). Notably, Target 16.2 of this goal directly mandates an end to all forms of violence against and torture of children (6, 7).

A holistic approach is essential for children to flourish within the sustainability paradigm. This method must address not only their immediate health and educational needs but also the broader socio-economic and environmental factors shaping their development. Child poverty, for example, remains a major socio-economic stressor at unacceptably high levels in many countries, although its impact can be moderated by the strength of national social protection systems (8). Some research suggests that achieving the SDGs is key to overcoming such socio-economic challenges, which would in turn enhance children’s well-being and leisure time (9, 10). Family and community support are central to this approach. Studies confirm that children from supportive environments who receive psychosocial or community-based support develop greater resilience and adaptive abilities (11). Educational and community programs that build skills for overcoming adversity can also effectively foster physical health, social skills, and emotional well-being (12, 13). It is critical, however, that these programs remain accessible to all, especially marginalized groups (14).

Given the interconnectedness of these issues, effective interventions depend on integrated policymaking. Such policies should adopt an ecological framework that acknowledges the complexity of children’s environments. This is the strategy that understands the multi-dimensional nature of child development and requires collaboration among health, education, and social services to increase the resilience of families and communities (15, 16). The comprehensive support system is very important for establishing the right conditions for the children to grow to their full potential.

Despite global consensus on its seriousness, child abuse and neglect (CAN) remains a widespread and often hidden phenomenon (17, 18). It represents a profound public health crisis and a severe breach of human rights, with consequences that extend far beyond immediate physical and emotional trauma. Long-term effects include a higher prevalence of chronic health problems, mental health disorders, educational failure, and reduced economic productivity in adulthood (17, 19, 20). This risk is not evenly distributed and is often exacerbated by psychosocial stressors, such as the trauma faced by vulnerable groups like refugee families (21). The prevalence of related mental health disorders is alarmingly high; for instance, one study found that 78% of adult survivors of child sexual abuse met the diagnostic criteria for PTSD at the start of their therapy (22). This cascade of adverse effects diminishes individual potential while imposing a heavy economic burden on public systems for healthcare, social welfare, and criminal justice (18, 20, 23).

However, this negative trajectory is not inevitable. A significant body of research focuses on ‘resilience,’ the capacity to adapt successfully in the face of adversity. Studies show that with key protective factors, many children who experience maltreatment avoid developing serious behavioral problems like aggression. These protective factors include individual characteristics, such as a child’s prosocial skills, as well as familial factors, like caregiver well-being (24). Without such protections, CAN can fuel a vicious intergenerational cycle of violence. Individuals who were abused in childhood are more likely to harm others, in turn damaging social harmony for future generations (25, 26).

While child abuse is commonly associated with direct acts of physical or sexual violence, child neglect is a quieter yet equally harmful phenomenon defined by omission. Neglect involves a caregiver’s continuous failure to provide for a child’s physical, emotional, educational, and medical needs (27). Some of the most insidious forms of maltreatment, like parental alienation, actually combine psychological abuse and emotional neglect, creating complex challenges for psychiatry, sociology, and justice systems (28). Because neglect is characterized by a failure to act rather than an intentional act of harm, it is much harder to identify, measure, and address legally. It is also deeply influenced by social factors like poverty and cultural parenting norms, which creates a complex web of interconnected issues that are difficult to resolve (29–31). Nevertheless, the harm caused by neglect is undeniable. It can severely damage a child’s developing brain and long-term well-being, leading to attachment disorders, developmental delays, and diminished resilience (32–34). Therefore, a sustainable child protection approach must be built on a comprehensive and vigilant understanding of all forms of both abuse and neglect (35).

To deconstruct the multifaceted nature of CAN and create effective interventions, a robust theoretical framework is paramount. Urie Bronfenbrenner’s Ecological Systems Theory offers such a framework (36, 37). This leading socio-ecological model is considered fundamental for understanding the complex interactions involved in CAN and for designing effective, multisectoral prevention strategies (38). The theory posits that human development is shaped by the interplay between an individual and a series of nested environmental systems of varying sizes. The microsystem comprises the child’s immediate settings, such as the family, school, and peer group. The mesosystem involves the connections between these microsystems, like the interaction between home and school life (39). The exosystem includes external environments that indirectly affect the child by influencing their caregivers, such as a parent’s workplace or community health services (40). Finally, the macrosystem encompasses the broader cultural values, laws, and norms that influence all other systems (41).

From an ecological perspective, CAN extends beyond a simple dysfunction in the parent–child relationship (microsystem). This microsystem-level dysfunction is particularly destructive because it fundamentally undermines the attachment process. The dynamic is insidious: parental rejection can trigger intense self-conscious emotions, causing a child to internalize profound shame and guilt, or to believe they are to blame for their own abandonment (42). When caregivers—meant to be a ‘secure base’—become a source of threat, the child may form an internal model of a hostile world, which in turn shapes their future emotional and behavioral responses (43). Therefore, CAN is better understood as a systemic failure, often marked by breakdowns at the mesosystem level: the interface between a family and its support organizations (44). The effectiveness of a society’s child protection network depends heavily on collaboration and a shared understanding among its various components (45). If these crucial connections are weak or misaligned, children at risk can be easily overlooked. This study focuses on this exact mesosystemic link by examining the preparedness of two professional groups whose collaboration is essential for building a reliable safety net for children.

Within the societal ecosystem, certain professionals act as “sentinels,” uniquely positioned to identify potential signs of abuse through their regular contact with children and families. Midwives and pre-school educators are two critical sentinel groups, each operating within distinct professional paradigms and engaging with families at different developmental stages.

Midwives are often a family’s first professional contact, providing care during the vulnerable antenatal, intrapartum, and postnatal periods. Focused primarily on the physical health of the mother and infant, their work allows them to observe parent-infant bonding, living conditions, and early indicators of physical neglect or maternal stress that could escalate to abuse (26, 46). Their perspective is therefore rooted in a medical model of care (46).

In contrast, pre-school educators have sustained, regular interaction with children in structured settings designed to foster cognitive, social, and emotional skills (47, 48). This long-term perspective allows them to observe a child’s behavior, peer interactions, and developmental trajectory. Consequently, they are well-positioned to recognize subtler signs of maltreatment, such as emotional abuse, developmental delays caused by neglect, or behavioral changes that may indicate trauma (49, 50). Their professional vision is shaped by pedagogy, child psychology, and developmental science (51–53).

Although both professions share the goal of ensuring child well-being, their distinct training and theoretical foundations can create different sensitivities and blind spots regarding CAN’s multifaceted nature. This divergence leads to a significant but under-researched problem: the “interprofessional awareness gap” (54, 55). This is not just a hypothetical risk between fields like health and education; it is a documented reality. For example, a recent study of midwives and physicians revealed fundamental differences in their perceptions of interprofessional collaboration. Midwives consistently rated teamwork and equitable communication significantly lower than their physician counterparts did (56). The authors attributed this perceptual divide to the “divergent professional backgrounds and fundamentally different approaches to care” within each profession. This finding supports our hypothesis that the awareness gap in CAN recognition stems from these foundational educational paradigms. If collaboration on maternal health is perceived so differently, then teamwork on a complex issue like child safeguarding becomes even more challenging without targeted interprofessional education. Ultimately, this gap creates variance in the knowledge and preparedness among professional groups for identifying different sub-types of child maltreatment. A midwife, for instance, might easily recognize signs of physical neglect but miss the developmental impact of an emotionally unstimulating home. Conversely, a teacher could spot learning difficulties from poor parental engagement but be unequipped to identify signs of medical neglect.

This awareness gap directly threatens the sustainability of child protection efforts. Effective safeguarding is not the sole responsibility of one agency; it depends on a unified, multi-agency approach that involves sharing resources and using a common vocabulary for risk and protection. When future health and education professionals enter their careers with vastly different perceptions of harm, the very foundation for interprofessional collaboration is weakened from the outset. This can lead to missed opportunities for early intervention, inconsistent responses to families in crisis, and a failure to establish the integrated support systems that vulnerable children urgently need. Therefore, the effectiveness of the entire child protection ecosystem hinges on a shared, in-depth understanding of the issue among its frontline professionals (57).

This investigation empirically explores the nature and scale of this potential awareness gap. Going beyond a general assessment, we used comprehensive, validated instruments to distinguish between physical, sexual, and emotional abuse. These tools also addressed distinct dimensions of neglect, such as the right to life and development and the rights of children with special needs. Ultimately, our aim is to analyze the specific strengths and vulnerabilities in each professional group’s preparedness. Understanding these nuanced differences is the crucial first step in developing educational interventions that can align the perspectives of these future professionals.

This study compares the preparedness of future midwives and pre-school educators in Türkiye to recognize various aspects of child abuse and neglect. By examining these two student groups before they enter their respective professions, we seek to understand how their university training shapes their foundational knowledge and perceptions. The Turkish context is particularly relevant because, despite existing laws, many child abuse and neglect cases go unreported to judicial authorities, highlighting a significant need for further research (58). Türkiye also serves as a useful case study, as its health and education professionals follow distinct but interconnected higher education pathways. Our findings may therefore inform conversations in other countries with similar systems. This paper concludes by discussing the vital role curriculum reform can play in fostering common ground for future interprofessional practice and a more integrated child protection system. Ultimately, preparing the next generation of sentinel professionals with a full understanding of their safeguarding duties is fundamental to strengthening the societal ecosystem and ensuring the sustainable well--being of every child.

### 1.1 Aim

This study’s primary aim is to compare the preparedness of final-year midwifery and pre-school education students in identifying the various forms of child abuse and neglect. Through this comparison, we seek to reveal specific gaps and overlaps in their awareness. Our findings are intended to provide an empirical basis for enhancing higher education curricula and fostering a more sustainable, collaborative child protection framework. To achieve this primary aim, the study pursues the following specific objectives:

1. To describe the overall awareness levels of child neglect and child abuse among midwifery and pre-school education students.
2. To compare the overall awareness scores for “child neglect” and “child abuse” between students of midwifery and pre-school education.
3. To conduct a granular analysis of potential differences in awareness across the specific sub-dimensions of child neglect.
4. To investigate the disparities in awareness across the distinct sub-dimensions of child abuse.
5. To examine the extent to which grade and prior formal “training” on the topic of CAN influence the awareness levels of students within both professional cohorts.

## 2 Materials and methods

This section outlines the methodological framework employed to investigate the interprofessional awareness gap. It details the causal-comparative research design, the characteristics of the participant sample, the validated psychometric instruments used for data collection, and the statistical procedures applied for data analysis. Each component was carefully selected to ensure a rigorous and systematic examination of the study’s core research questions.

### 2.1 Research design

This study employs a non-experimental, quantitative causal-comparative (ex post facto) design to examine university students’ awareness of child abuse and neglect (59). This approach is ideal for comparing differences in a dependent variable (CAN awareness) between two pre-existing, naturally formed groups: midwifery and pre-school education students (60). Instead of manipulating the independent variable (students’ academic department), we examined its potential connection to the outcome variable after the fact. This design directly addresses our central research questions about the “interprofessional awareness gap” by allowing for a systematic comparison of the two groups’ mean scores across all scales and sub-dimensions. While this non-experimental design cannot definitively establish causality like a true experiment (59, 60), it is highly effective for exploring potential influences and identifying significant differences within an authentic academic setting. By examining how different educational pathways influence preparedness, this design provides the insights necessary to inform evidence-based curriculum development and promote a sustainable, integrated child protection system.

### 2.2 Participants

The study’s population was made up of undergraduate students from a public university in Türkiye. The research sampling unit was the individual undergraduate student in two specific ‘sentinel professions,’ i.e., midwifery and pre-school education. The formal sampling frame was constructed by all students enrolled in these two departments during the period of data collection.

A criterion non-probability sampling design was chosen for recruiting the research participants. Specifically, a convenience sampling method was implemented, which was focused on students who were available and accessible during data collection periods. Such a method does not allow statistical generalization of the total student population in Türkiye but the purposive selection of these two specific academic departments was crucial for addressing the study’s main research question about the “interprofessional awareness gap.” This approach ensured the sample was the most applicable as it was directly relevant to the study’s aim of comparing future professionals which will be holding the key safeguarding responsibilities. Participation was anonymous and voluntary.

The number of students reached in the final study was 361. The sufficiency of sample size is of great importance in ex post facto and causal-comparative research, where the aim is to provide enough statistical power and accurately compare groups. The methodological literature that is widely accepted and used as a reference supports the idea that for these types of research at least 30 participants per group should be included to make meaningful statistical analyses possible (60). The group sizes in this study with 246 students from the midwifery department and 115 from pre-school education exceed this limit with a big margin. Following the statistical power rules, this strong sample size contributes to the detection of the actual difference between the groups if at least one exists and also to the stabilization of the results at a high level of confidence (59, 61). Thus, the sample was considered large enough and appropriate for the planned inferential statistical tests such as t-tests and multivariate analyses of variance. Table 1 provides the detailed demographic composition of the sample.

**Table 1:** Demographic characteristics of the participants (*N* = 361).

Characteristic	Category	Frequency (f)	Percentage (%)
Department	Midwifery	246	68.1
	Pre-school education	115	31.9
Age group	18–20 years	206	57.1
	21–23 years	134	37.1
	24 years and above	21	5.8
Grade	1st Year	61	16.9
	2nd Year	108	29.9
	3rd Year	103	28.5
	4th Year	89	24.7
Prior training on CAN	Yes	225	62.3
	No	136	37.7
Place of residence	Province center	138	38.2
	District center	158	43.8
	Village	65	18.0
Graduated high school	Anatolian high school	267	74.0
	Science high school	64	17.7
	Health vocational HS	30	8.3

The details of the demographic composition of the sample are provided in Table 1. The participants were selected from two different educational programs, among which the number of representatives from the midwifery department was higher (68.1%) in comparison with those from the pre-school education department (31.9%). Such a distribution is the ground for the causal-comparative analysis of the study. The ages of the participants are a reflection of a normal undergraduate population, with almost all of them aged between 18–23 years old (94.2%).

All four years of academic study were represented, hence, it was possible to analyze the potential impact of educational progression on the awareness level. The largest groups were second-year students (29.9%) and third-year students (28.5%). A most notable feature of the sample is that the majority of the participants (62.3%) claimed that they had got some training related to child abuse and neglect in the past. The students were mostly educated in Anatolian High School (74.0%), and the participants were from different types of places, with the largest number coming from district centers (43.8%).

### 2.3 Data collection instruments

In order to collect the required data for the research, two validated psychometric instruments, the Child Neglect Awareness Scale, and the Child Abuse Awareness Scale were implemented. Both scales were created in Turkish by Celiloğlu and Özer Aytekin (62) solely for the purpose of measuring the awareness levels of the future educational staff members, who are going to work with kids, e.g., pre-school teacher candidates. These tools were chosen because of their strong psychometric qualities and their close connection with the aims of the research.

#### 2.3.1 Child Neglect Awareness Scale

The Child Neglect Awareness Scale created by Celiloğlu and Özer Aytekin (62) aimed at raising the awareness of the participants about different types of child neglect was the tool used. The instrument aims to go beyond the general understanding of neglect by delving into specific dimensions which are based on children’s rights. The researchers here considered the total score of neglect as well as those aspects that were mentioned in the literature, such as the “Right to Life and Development” and the “Rights of Children with Special Needs”.

The scale’s items describe the different situations and the conditions of the subjects (e.g., “Failure to take necessary precautions for a child with a visual impairment before they go outside”), and the participants are required to specify the degree of their concurrence with the statement that the situation is neglect. On a scale of 1–5, respondents indicate their level of agreement to the respective statement with 1 (Strongly Disagree) and 5 (Strongly Agree) as the extreme points of the scale. The higher the mean score on the scale and its sub-dimensions is, the higher the awareness of child neglect is considered. The original study on the validation of the scale revealed the strong psychometric properties of the scale, which was confirmed by factor analysis for the construct validity, and a high internal consistency was reported, with a Cronbach’s alpha coefficient of 0.92 for the whole scale, being the latter indicator. The scale also demonstrated excellent internal consistency in the present study, with a calculated Cronbach’s alpha coefficient of 0.97.

#### 2.3.2 Child Abuse Awareness Scale

Participants’ awareness of child abuse was assessed with the Child Abuse Awareness Scale, which was also created by Celiloğlu and Özer Aytekin (62). This scale aims to measure the capability of future professionals to identify the behavioral and situational figures of the different types of child abuse. The tool is organized into sub-dimensions that represent the different categories of maltreatment, such as Physical Abuse, Sexual Abuse, Emotional Abuse, and Economic Abuse. The way that the neglect scale is constructed, participants may read the descriptions of the specific child behaviors or situations (e.g., “A child frequently coming to school with cutting/piercing tools”) and react by selecting a rating on the 5-point Likert-type scale (1 = Strongly Disagree; 5 = Strongly Agree). The higher the scores, the more the signs and the different forms of child abuse familiar to the participants. The validation research has substantiated the reliability, with a Cronbach’s alpha coefficient of 0.86 being reported for the overall scale, which is indicative of excellent internal consistency. In the present study, a reliability analysis confirmed the scale’s excellent internal consistency for the current sample, yielding a Cronbach’s alpha coefficient of 0.95 for the overall scale. Scale is identified as valid and reliable instruments for measuring the level of preparedness of the sample in this study.

### 2.4 Data analysis

IBM SPSS Statistics were used to conduct all statistical analyses. The alpha level for determining statistical significance was set at *p* < 0.05. To begin with, awareness of the sample to profile, descriptive statistics (means and standard deviations) were calculated for the overall scores and sub-dimensions of the child neglect and abuse scales for both student groups.

In order to meet the specific research objectives, i mplemented a set of inferential tests. Firstly, Independent Samples t-tests compared the overall mean awareness scores for total child neglect and total child abuse between midwifery and pre-school education students. Secondly, to perform a more detailed comparison, two separate one-way multivariate analyses of variance (MANOVA) were run to test the differences between the two departments in the sub-dimensions of child neglect and the sub-dimensions of child abuse, respectively.

Moreover, to study the impact of academic progression, as well as that of prior training, two three-way analyses of variance (ANOVAs) were carried out. The academic department, prior training status, and grade were the independent variables in these models, while the total neglect and total abuse awareness scores were the dependent variables in separate analyses. The assumptions for parametric testing, such as the homogeneity of variances determined by Levene’s Test, were confirmed for all the tests.

## 3 Results

The statistical results of the study are being presented in this section. The discussion starts with the descriptive results that depict the basic understanding of the awareness of child abuse and neglect among midwifery and pre-school education students. This primary survey offers a basic understanding of the topic before going further with the comparison of the two professional groups by means of inferential analyses.

### 3.1 Descriptive analysis of child abuse and neglect awareness levels

The initial analysis focused on establishing a descriptive profile of each professional cohort’s awareness. Table 2 summarizes these foundational statistics, presenting the mean scores and standard deviations for all measured variables for both the midwifery and pre-school education groups.

**Table 2:** Descriptive statistics for awareness of child neglect and abuse by academic department.

Variable	Midwifery students (*n* = 246)	Pre-school education students (*n* = 115)
	M (SD)	M (SD)
Total child neglect	4.37 (0.52)	4.52 (0.43)
Child neglect	4.28 (0.51)	4.40 (0.43)
Right to life and development	4.43 (0.58)	4.59 (0.46)
Rights of children with special needs	4.39 (0.60)	4.56 (0.51)
Total child abuse	3.99 (0.54)	4.15 (0.48)
Physical abuse	4.01 (0.67)	4.13 (0.67)
Sexual abuse	3.72 (0.78)	3.83 (0.76)
Emotional abuse	4.11 (0.57)	4.33 (0.48)
Economic abuse	4.13 (0.72)	4.32 (0.63)

M, mean; SD, standard deviation. Scores were measured on a 5-point Likert scale, with higher scores indicating greater awareness.

Table 2 presents the descriptive statistics for the awareness of child neglect and abuse among students in midwifery and pre-school education programs. The findings indicate that, overall, participants from both departments demonstrated a high level of awareness regarding child maltreatment, with all mean scores positioned well above the midpoint of the 5-point scale.

For midwifery students, the mean awareness score for total child neglect was high (*M* = 4.37, SD = 0.52). Among the neglect sub-dimensions, their highest awareness was related to the “Right to Life and Development” (*M* = 4.43, SD = 0.58). Their awareness of total child abuse was also notably high (*M* = 3.99, SD = 0.54), with the highest scores in “Economic Abuse” (*M* = 4.13, SD = 0.72) and “Emotional Abuse” (*M* = 4.11, SD = 0.57). The lowest mean score for this group was observed in the “Sexual Abuse” sub-dimension (*M* = 3.72, SD = 0.78).

Similarly, pre-school education students displayed a very high level of awareness. Their mean score for total child neglect was (*M* = 4.52, SD = 0.43), and for total child abuse, it was (*M* = 4.15, SD = 0.48). This cohort also scored highest on the “Right to Life and Development” sub-dimension of neglect (*M* = 4.59, SD = 0.46). As with the midwifery students, the pre-school education students’ lowest mean awareness score was in the area of “Sexual Abuse” (*M* = 3.83, SD = 0.76).

While these scores are commendably high, it is critical to address that they do not approach the maximum value of 5.00. For future sentinel professionals who will be responsible for the safeguarding of children, any gap between “high” and “expert” awareness represents a potential risk. The fact that awareness scores are not ceilinged suggests that there are still nuances of neglect and abuse that a portion of students may not fully recognize. This is particularly concerning for the “Sexual Abuse” sub-dimension, which consistently scored the lowest for both groups. This discrepancy highlights a critical area for improvement in professional training, suggesting that while foundational knowledge is strong, curricula must be enhanced to ensure a comprehensive and unequivocal understanding of all facets of child maltreatment.

### 3.2 Interprofessional comparison of overall awareness in child abuse and neglect

To investigate the potential “interprofessional awareness gap,” an Independent Samples t-test was conducted to compare the mean awareness scores of midwifery and pre-school education students. The analysis focused on the total scores for both child neglect and child abuse. Levene’s Test for Equality of Variances indicated that equal variances could be assumed for both child neglect (*p* = 0.063) and child abuse (*p* = 0.259). The results of the t-test, presented in Table 3, show statistically significant differences between the two student groups on both measures.

**Table 3:** Results of independent samples *t*-test for overall awareness scores by academic department.

Scale	Academic department	N	M (SD)	*t*	*df*	*p*
Total child neglect awareness	Midwifery	246	4.37 (0.52)	−2.700	359	**0.007**
	Pre-school education	115	4.52 (0.43)			
Total child abuse awareness	Midwifery	246	3.99 (0.54)	−2.681	359	**0.008**
	Pre-school education	115	4.15 (0.48)			

M, mean; SD, standard deviation. N, number of participants. *p*-values indicate statistical significance at *p* < 0.01. Bold values indicate statistical significance (*p* < 0.05).

The analysis reveals a significant disparity in the awareness levels of the two future professional cohorts.

For Total Child Neglect Awareness, there was a statistically significant difference in the scores for midwifery students (*M* = 4.37, SD = 0.52) and pre-school education students (*M* = 4.52, SD = 0.43); t(359) = −2.700, *p* = 0.007. This result indicates that students in the pre-school education program possess a significantly higher level of awareness regarding child neglect compared to their counterparts in the midwifery program.

A similar statistically significant finding was observed for Total Child Abuse Awareness. Pre-school education students (*M* = 4.15, SD = 0.48) again demonstrated a significantly higher level of awareness than midwifery students (*M* = 3.99, SD = 0.54); t(359) = −2.681, *p* = 0.008.

These findings provide robust empirical evidence for the existence of an interprofessional awareness gap between these two groups of future professionals. For both child neglect and child abuse, students of pre-school education showed a more developed awareness than midwifery students. This suggests that variations in their respective university curricula and educational focus likely contribute to different levels of preparedness for identifying and understanding the complexities of child maltreatment.

### 3.3 Comparative analysis of awareness across sub-dimensions of child neglect

To conduct a granular analysis of the interprofessional awareness gap, a one-way multivariate analysis of variance (MANOVA) was performed. This analysis examined the effect of the academic department (midwifery vs. pre-school education) on the three distinct sub-dimensions of child neglect: Child Neglect, Right to Life and Development, and Rights of Children with Special Needs.

The initial multivariate test, which assesses the combined effect on all dependent variables, was not statistically significant, though it approached the significance threshold (Wilks’ *Λ* = 0.980, *F*(3, 357) = 2.437, *p* = 0.064). While this overall result suggests that the combined dependent variables do not significantly differ across groups, it is crucial to examine the follow-up univariate analyses to explore potential differences within each specific sub-dimension. The detailed results of these tests, including descriptive statistics and effect sizes, are presented in Table 4.

**Table 4:** Descriptive statistics and univariate analysis of variance (ANOVA) for child neglect sub-dimensions by academic department.

Dependent variable	Academic department	*N*	M (SD)	*F*	*df*	*p*	Partial η^2^
Child neglect	Midwifery	246	4.28 (0.51)	5.269	1, 359	**0.022**	0.014
	Pre-school education	115	4.40 (0.43)				
Right to life and development	Midwifery	246	4.43 (0.58)	6.576	1, 359	**0.011**	0.018
	Pre-school education	115	4.59 (0.46)				
Rights of children with special needs	Midwifery	246	4.39 (0.60)	6.781	1, 359	**0.010**	0.019
	Pre-school education	115	4.56 (0.51)				

M, mean; SD, standard deviation; *N*, number of participants; Partial η^2^, partial eta squared. *p*-values indicate statistical significance at *p* < 0.05. Bold values indicate statistical significance (*p* < 0.05).

Although the omnibus MANOVA test did not yield a statistically significant result, the detailed univariate analyses provide a more granular and insightful perspective, revealing a consistent pattern of significant differences across all individual sub-dimensions of child neglect.

For the “Child Neglect” sub-dimension, a statistically significant difference was found between the two groups, *F*(1, 359) = 5.269, *p* = 0.022. Pre-school education students (*M* = 4.40, SD = 0.43) reported a significantly higher awareness level than midwifery students (*M* = 4.28, SD = 0.51). The effect size for this difference was small (Partial η^2^ = 0.014), indicating that academic department accounted for 1.4% of the variance in these scores.

Similarly, for the “Right to Life and Development” sub-dimension, there was a significant difference, *F*(1, 359) = 6.576, *p* = 0.011. The scores of pre-school education students (*M* = 4.59, SD = 0.46) were significantly higher than those of midwifery students (*M* = 4.43, SD = 0.58). The effect size was again small (Partial η^2^ = 0.018), explaining 1.8% of the variance.

This pattern was also evident in the “Rights of Children with Special Needs” sub-dimension, which showed a significant difference, F(1, 359) = 6.781, *p* = 0.010. Once more, pre-school education students (*M* = 4.56, SD = 0.51) demonstrated significantly greater awareness than midwifery students (*M* = 4.39, SD = 0.60). This variable had the largest, albeit still small, effect size (Partial *η*^2^ = 0.019), with the academic program explaining 1.9% of the variance in awareness scores.

The granular analysis powerfully demonstrates that despite the borderline multivariate result, a clear and consistent interprofessional awareness gap exists. Pre-school education students consistently show a significantly higher level of awareness across every measured facet of child neglect. This finding not only supports the result from the second objective regarding overall neglect awareness but also pinpoints that this gap is not confined to a single area; rather, it is a pervasive difference that spans multiple, critical dimensions of child protection knowledge.

### 3.4 Comparative analysis of awareness across sub-dimensions of child abuse

To assess the interprofessional awareness gap across the different facets of child abuse, a one-way multivariate analysis of variance (MANOVA) was conducted. The analysis aimed to determine the effect of the academic department (midwifery vs. pre-school education) on awareness levels across four sub-dimensions: Physical Abuse, Sexual Abuse, Emotional Abuse, and Economic Abuse.

The results showed a statistically significant multivariate main effect for the academic department, Wilks’ *Λ* = 0.962, *F*(4, 356) = 3.549, *p* = 0.007. This indicates that there is a significant overall difference between midwifery and pre-school education students when their awareness levels on the four types of abuse are considered together.

To pinpoint the specific sub-dimensions contributing to this overall significant difference, the results of the follow-up univariate analyses (Tests of Between-Subjects Effects) were examined. These detailed results are presented in Table 5.

**Table 5:** Descriptive statistics and univariate analysis of variance (ANOVA) for child abuse sub-dimensions by academic department.

Dependent variable	Academic department	N	M (SD)	F	*df*	*p*	Partial η^2^
Physical abuse	Midwifery	246	4.01 (0.67)	2.260	1, 359	0.134	0.006
	Pre-school education	115	4.13 (0.67)				
Sexual abuse	Midwifery	246	3.72 (0.78)	1.595	1, 359	0.207	0.004
	Pre-school education	115	3.83 (0.76)				
Emotional abuse	Midwifery	246	4.11 (0.57)	13.062	1, 359	**0.000**	0.035
	Pre-school education	115	4.33 (0.48)				
Economic abuse	Midwifery	246	4.13 (0.72)	5.910	1, 359	**0.016**	0.016
	Pre-school education	115	4.32 (0.63)				

M, mean; SD, standard deviation; *N*, number of participants; Partial η^2^, partial eta squared. *p*-values indicate statistical significance at *p* < 0.05. Bold values indicate statistical significance (*p* < 0.05).

The follow-up univariate tests reveal a nuanced picture, indicating that the significant overall difference in child abuse awareness is driven by specific sub-dimensions, while others show no significant variation between the groups.

Emotional Abuse: The most substantial difference was found in the awareness of emotional abuse, *F*(1, 359) = 13.062, *p* < 0.001. Pre-school education students (*M* = 4.33, SD = 0.48) demonstrated a significantly higher level of awareness than midwifery students (*M* = 4.11, SD = 0.57). The effect size for this difference was moderate (Partial η^2^ = 0.035), indicating that the academic department accounted for 3.5% of the variance in awareness scores for emotional abuse.

Economic Abuse: A statistically significant difference was also observed in the awareness of economic abuse, F(1, 359) = 5.910, *p* = 0.016. Consistent with the previous finding, pre-school education students (*M* = 4.32, SD = 0.63) scored significantly higher than midwifery students (*M* = 4.13, SD = 0.72). The effect size for this difference was small (Partial η^2^ = 0.016).

Physical and Sexual Abuse: In contrast, the analyses for physical abuse (*p* = 0.134) and sexual abuse (*p* = 0.207) showed no statistically significant differences between the two groups. Although the mean scores of pre-school students were slightly higher, this variation was not sufficient to reach statistical significance.

In summary, the granular analysis of child abuse awareness reveals a specific and targeted interprofessional gap. The overall difference between future midwives and pre-school educators is not uniform across all categories of abuse. Instead, it is primarily driven by the significantly higher awareness of pre-school education students regarding emotional and economic abuse. Both professional cohorts appear to have statistically comparable levels of awareness concerning the more traditionally recognized forms of physical and sexual abuse.

### 3.5 The influence of grade and prior training on child neglect awareness

A three-way analysis of variance (ANOVA) was conducted to examine the influence of academic department, prior training, and grade on students’ awareness of child neglect. The analysis also sought to identify any potential interaction effects between these factors. The assumption of homogeneity of error variances was met, as assessed by Levene’s Test of Equality of Error Variances, *F*(15, 345) = 1.400, *p* = 0.144.

The results of the ANOVA, summarized in Table 6, indicate a significant main effect for the academic department, but no significant main effects for prior training or class year. Furthermore, none of the interaction effects reached statistical significance at the *p* < 0.05 level.

**Table 6:** Results of Three-Way ANOVA for Factors Influencing Child Neglect Awareness.

Source	*df*	*F*	*p*	Partial η^2^
Academic Department	1	7.489	**0.007**	0.021
Prior Training	1	1.618	0.204	0.005
Grade	3	1.992	0.115	0.017
Department * Training	1	3.115	0.078	0.009
Department * Grade	3	0.256	0.857	0.002
Training * Grade	3	2.581	0.053	0.022
Department * Training * Grade	3	0.927	0.428	0.008

Dependent variable: Total child neglect awareness. The error term for F-tests is mean square (Error) = 0.235 with df = 345. *p*-value indicates statistical significance. Bold values indicate statistical significance (*p* < 0.05).

A detailed examination of the main effects and interactions provides a nuanced understanding of the factors shaping child neglect awareness. The estimated marginal means for the significant and borderline effects are presented in Table 7.

**Table 7:** Estimated marginal means for child neglect awareness.

Effect	Factor level	Mean	Std. Error	95% Confidence interval
				Lower
Academic department	Midwifery	4.339	0.033	4.274
	Pre-school education	4.505	0.051	4.405
Training * Grade	Prior training: Yes			
	1st Year	4.487	0.078	4.334
	2nd Year	4.458	0.063	4.334
	3rd Year	4.436	0.073	4.293
	4th Year	4.461	0.075	4.313
	Prior training: No			
	1st Year	4.405	0.136	4.137
	2nd Year	4.262	0.082	4.101
	3rd Year	4.599	0.074	4.454
	4th Year	4.267	0.084	4.103

Means are derived from the ANOVA model.

#### 3.5.1 Main effects

The analysis revealed a statistically significant main effect for Academic Department, *F*(1, 345) = 7.489, *p* = 0.007, with a small effect size (Partial η^2^ = 0.021). Consistent with previous findings, pre-school education students (*M* = 4.505) demonstrated a significantly higher awareness of child neglect than midwifery students (*M* = 4.339).

However, there was no significant main effect for Prior Training, F(1, 345) = 1.618, *p* = 0.204. This indicates that, when considered across all students, having previously received training on child abuse and neglect did not result in a statistically significant difference in overall neglect awareness. Similarly, the main effect for Grade was not significant, *F*(3, 345) = 1.992, *p* = 0.115, suggesting that awareness of child neglect does not systematically increase or decrease as students progress through their academic programs.

#### 3.5.2 Interaction effects

None of the interaction effects were statistically significant at the 0.05 level. However, the Training * Grade interaction approached statistical significance, F(3, 345) = 2.581, *p* = 0.053, Partial η^2^ = 0.022. The pattern of this interaction, illustrated in the provided plot, is noteworthy. For students who had prior training, awareness levels remained consistently high and stable across all four academic years. In contrast, for students with no prior training, awareness levels were more volatile: they were relatively high in the 1st year, dipped in the 2nd year, peaked sharply in the 3rd year, and dropped again in the 4th year. This suggests that academic progression may have a different, more inconsistent impact on awareness for students who lack a foundational training background.

The remaining two-way and three-way interactions were not significant (*p* > 0.05).

In summary, the most robust and significant predictor of child neglect awareness among the factors examined is the student’s academic department. The influences of prior training and year of study were not significant on their own, but the borderline interaction between them suggests a complex relationship where the educational journey’s impact on awareness may depend on whether students have previously received formal training.

### 3.6 The influence of grade and prior training on child abuse awareness

A three-way analysis of variance (ANOVA) was conducted to investigate the main and interactive effects of academic department, prior training, and grade on students’ awareness of child abuse. The assumption of homogeneity of variances was met, as confirmed by Levene’s Test (*p* = 0.885).

The ANOVA results, displayed in Table 8, revealed a statistically significant main effect for academic department. However, no significant main effects were found for prior training or grade. Furthermore, none of the two-way or three-way interactions between the factors were statistically significant.

**Table 8:** Results of three-way ANOVA for factors influencing child abuse awareness.

Source	*df*	*F*	*p*	Partial η^2^
Academic department	1	8.446	**0.004**	0.024
Prior training	1	1.380	0.241	0.004
Grade	3	1.788	0.149	0.015
Department * Training	1	0.184	0.668	0.001
Department * Grade	3	0.111	0.954	0.001
Training * Grade	3	0.888	0.447	0.008
Department * Training * Grade	3	1.692	0.168	0.014

Dependent variable: total child abuse awareness. The error term is mean square (Error) = 0.272 with df = 345. The *p*-value indicates statistical significance. Bold values indicate statistical significance (*p* < 0.05).

The analysis points to the student’s academic department as the sole significant factor influencing child abuse awareness among the variables tested. The estimated marginal means for this significant effect are presented in Table 9.

**Table 9:** Estimated marginal means for child abuse awareness by academic department.

Academic Department	Mean	Std. Error	95% Confidence interval
			Lower
Midwifery	3.974	0.036	3.903
Pre-school Education	4.163	0.055	4.056

Means are derived from the ANOVA model.

#### 3.6.1 Main effects

A statistically significant main effect was found for Academic Department, *F*(1, 345) = 8.446, *p* = 0.004, with a small effect size (Partial η^2^ = 0.024). This result confirms that pre-school education students (*M* = 4.163) possess a significantly higher level of awareness regarding child abuse compared to midwifery students (*M* = 3.974).

In contrast, the main effect for Prior Training was not statistically significant, F(1, 345) = 1.380, *p* = 0.241. This suggests that having previously received formal training on the topic does not, on its own, correspond to a significant difference in overall child abuse awareness levels in this sample. Similarly, the main effect for Grade was not significant, *F*(3, 345) = 1.788, *p* = 0.149, indicating that awareness does not significantly change as students progress from their first to their final year of study.

#### 3.6.2 Interaction effects

The analysis revealed no significant two-way or three-way interactions between the independent variables. The non-significant interaction between Department and Training (*p* = 0.668), for example, indicates that the effect of having prior training is not significantly different for midwifery and pre-school education students. Likewise, the non-significant interaction between Department and Grade (*p* = 0.954) suggests that the developmental trajectory of awareness across the four years is similar for both departments.

In summary, the findings for child abuse awareness are clear and direct. The academic department a student is enrolled in is the only significant predictor of their awareness level. Pre-school education students demonstrate a higher awareness than midwifery students, and this difference remains consistent regardless of their year of study or whether they have received prior training on the subject.

## 4 Discussion

This study analyzed the preparedness of future midwives and pre-school educators—two key sentinel professions—in identifying child abuse and neglect (CAN). Our findings provide robust empirical evidence for a significant “interprofessional awareness gap,” a disparity with profound implications for building a resilient and sustainable child protection system. In this discussion, we will contextualize these findings and argue that this gap represents a critical vulnerability in the societal ecosystem designed to protect children, ultimately compromising social sustainability and the long-term development of human capital.

### 4.1 The professional lens: disciplinary roots of the awareness gap

A major and consistent finding from this research is that early childhood education students possess significantly greater knowledge of Child Abuse and Neglect (CAN) than their midwifery counterparts. We attribute this disparity not to a difference in professional dedication, but to the systemic outcomes of their distinct educational paradigms—their ‘professional lenses’.

Pre-school education curricula are grounded in child development theories, pedagogy, and socio-emotional learning. This training helps educators develop a heightened awareness of the behavioral, developmental, and relational cues of maltreatment, including subtler forms like emotional abuse and neglect (63, 64). Such relational cues are critical, as they can manifest in social avoidance or submissive behaviors, often reflecting the deep-seated shame and guilt that children internalize after parental neglect or abandonment (42). Beyond their formal education, the personal histories of these professionals also warrant investigation. Research on pre-school teacher candidates, for instance, highlights the risk that educators who experienced childhood abuse may replicate similar behaviors with children under their care, making it imperative to break this cycle (65). This increased awareness among teachers is reflected in official reporting data. A comprehensive study by Gilbert et al. (66) found that educational staff are the single largest source of professional referrals to child protection services in countries like the USA, accounting for 16.5% of all reports. This figure is substantially higher than reports from medical staff, which constitute only 8.4%. These reporting patterns align with our study’s findings, suggesting the education sector’s focus on child development fosters a greater willingness to recognize and report suspected cases of CAN.

This professional lens appears to cause educators to focus on specific aspects of CAN. For instance, a study on preschool teachers in Türkiye revealed that while they acknowledged the general issue, they tended to define abuse primarily as ‘physical abuse’ and neglect as ‘emotional neglect’ (58). This suggests that even with training in child development, their interpretation of harm is often filtered through the most recognizable forms within their professional context. This finding aligns with observations from Schols et al. (67), who also noted that professional training leads to distinct differences in attitude and communication skills between teachers and healthcare professionals. Because their training involves long-term observation of a child’s total development, educators are better equipped to identify subtle deviations from developmental norms that may signal underlying trauma or deprivation.

Midwifery education is largely based on a biomedical model focused on the mother and infant’s physical health during the perinatal period. While this approach is useful for identifying physical neglect and abuse, research shows that reporting is a complex task. For example, a study by Flaherty et al. (68) found that primary care physicians failed to report 27% of cases despite a high suspicion of physical abuse, a hesitation influenced by injury characteristics, family risk factors, and personal experiences. Educators face a similar challenge. Despite high awareness, they often encounter a ‘conflict of loyalty,’ balancing their reporting duty against the need to maintain a trusting relationship with the child and parents. This dilemma is intensified in traditional communities; a study of kindergarten teachers in Israel described it as ‘entrapment’ between state regulations and cultural codes that prioritize family secrecy, which can even lead to threats against the teacher (69). Such conflicts, combined with a fear of causing more harm or a lack of trust in authorities, create reporting barriers that extend far beyond a simple knowledge gap (70).

Despite these shared challenges, midwifery training does not typically equip professionals with the same level of competency as pre-school educators in identifying subtle psycho-social signs of distress or neglect. The German “Family Midwife” program tacitly acknowledges this shortcoming. In this program, skilled midwives receive additional qualifications to provide continuous, home-visiting support to disadvantaged families, filling a gap left by standard short-term medical and social services (71). The existence of such a program suggests that the traditional midwifery role may be insufficient for tackling neglect in high-risk environments. This need for enhanced psycho-social training is further underscored by the intergenerational transmission of risk. Research shows that parental behaviors like excessive alcohol intake and depression are strong predictors of similar issues in their adolescent children (72). A midwife operating within a strict biomedical framework may overlook these parental risk factors, missing a critical opportunity for early intervention. This is particularly significant because medical referrals are highly credible to child welfare systems; investigations initiated by medical professionals are over three times more likely to result in ongoing services (73). Therefore, the awareness gap in midwifery is not just a single missed report; it is a systemic barrier that prevents vulnerable families from accessing the intensive, long-term support that a medical referral can uniquely unlock.

### 4.2 Deconstructing the gap: divergence in emotional and economic abuse awareness

A detailed analysis reveals that the awareness gap is not uniform. While both professional groups showed similar knowledge of physical and sexual abuse—forms commonly addressed in public campaigns and training—a significant disparity emerged regarding emotional and economic abuse. The gap in recognizing economic abuse is particularly concerning because this form of maltreatment is often overlooked. As a study by Bruno (74) highlights, economic abuse has traditionally been subsumed under psychological abuse, causing its unique characteristics to go unrecognized. It can involve controlling a person’s access to economic resources, impacting children both indirectly through parental hardship and directly when their own property or savings are taken (74).

The consequences of these less visible forms of abuse are severe, representing direct assaults on the formation of human capital—the foundation of a sustainable society. For instance, recent neuroscientific research links childhood emotional and physical abuse to dysfunction in the brain’s automatic emotion regulation processes (75). Such abuse can interrupt neural circuits essential for emotional management, explaining its lasting negative impact. However, recognizing these subtle forms of harm is a major challenge. Research on preschool teachers in Estonia, for example, found that while they understood maltreatment generally, they found emotional abuse and neglect significantly harder to identify than physical abuse, which led to a lack of confidence in reporting (76).

The higher awareness among pre-school educators in these areas is logical. Their profession requires them to monitor the long-term consequences of non-stimulating or emotionally toxic home environments, which can lead to developmental delays, attachment disorders, and impaired social functioning. In contrast, midwifery students typically have intensive yet short-term, medically focused interactions with families, making them less familiar with the gradual impact of these less visible forms of maltreatment. This lack of familiarity is concerning because complex issues like parental alienation—a form of psychological abuse that is difficult to objectify—are known to have severe long-term psychosocial consequences, including depression, anxiety, substance abuse, and a profound lack of trust in adulthood (28). This finding underscores a critical point: an effective child protection strategy must move beyond reacting to overt harm and develop a deeper understanding of how unmet needs and emotional hostility compromise a child’s future opportunities.

### 4.3 Beyond silos: systemic implications for a sustainable protection ecosystem

This research is framed by Bronfenbrenner’s Ecological Systems Theory, which posits that child development emerges from interactions within hierarchically arranged systems (36). Our findings reveal a significant breakdown at the mesosystem level—specifically at the interface between the family and key institutions like health and education. This ‘awareness gap’ suggests that professionals in these sectors may lack a shared language or a common understanding of risk, leading to inconsistent support for vulnerable families and missed opportunities for intervention. This mesosystemic failure is particularly critical as it prevents intervention at the foundational level of the Adverse Childhood Experiences (ACEs) pyramid. The ACEs framework demonstrates how early adversity can lead to a cascade of negative outcomes, including neurodevelopmental disruptions, health-risk behaviors, and ultimately, disease, disability, or early death (2). Therefore, this interprofessional awareness gap is not simply an educational shortcoming; it is a serious public health vulnerability that allows this damaging sequence of events to continue unchecked.

This systemic disparity directly impacts the progress toward the United Nations’ 2030 Agenda. Specifically, the awareness deficit in future midwives hinders the proactive health-monitoring mandates of SDG 3 (Good Health and Well-being), as unrecognized maltreatment remains a leading cause of long-term morbidity. Conversely, the higher awareness among pre-school educators underscores the potential of the education sector to act as a safeguard for SDG 4 (Quality Education) by ensuring safe, inclusive learning environments. Ultimately, reconciling these professional lenses is not merely a curricular issue but a structural necessity for fulfilling Target 16.2, which demands ending all forms of violence against children through resilient and integrated institutional frameworks.

Our investigation revealed that a student’s academic department was the sole significant predictor of awareness, surpassing the influence of prior training or grade level. This finding underscores the paramount importance of a professional’s foundational ‘lens,’ which is shaped by their core university curriculum. Without a holistic, socio-ecological perspective integrated into their training, professionals may be unable to recognize nuanced risk factors like the acculturative stress and weakened family functionality seen in refugee parents (21). Our results align with a study of teachers in Spain, which found no significant difference in child abuse knowledge between trainees and experienced teachers, suggesting that professional experience did not compensate for gaps in foundational training (77). This appears to be a systemic issue. Similarly, a study of dental students found that although most were aware of CAN, their formal curriculum was their least-used information source. That study also concluded that without specific training, students felt ‘powerless’ despite their awareness—a finding that parallels our conclusion about the foundational professional lens being the critical determinant of preparedness (78). Ultimately, these findings collectively suggest that on-the-job experience cannot replace a deficient foundational education.

This result is supported by worldwide research, which shows that despite the proven benefits of focused pre-service training, many professionals start their careers unprepared due to irregular and fragmented education (79). The severe consequences of this gap are reflected in the testimonies of abuse survivors. These survivors often report that educators mislabeled their behavioral changes as disciplinary issues rather than cries for help, and they explicitly call for mandatory, comprehensive training as a critical tool for prevention and intervention (80). This lack of formal, discipline-specific knowledge forces professionals to become resourceful on the job. For example, a qualitative study on early childhood teachers in Australia found that in the absence of specific training on CAN, teachers had to adapt their general pedagogical knowledge to meet the child protection challenges they encountered.

While this adaptive competency is notable, it also exposes significant gaps in foundational knowledge, highlighting an indispensable need for formal training (81). This indicates that standalone workshops and passive engagement with curricula are insufficient to bridge this knowledge chasm. The root of the problem appears to be the very design and content of the foundational university programs themselves. This inference is supported by research across various fields. For example, a recent study of nursing students in Jordan directly linked their lack of knowledge about CAN to a curriculum that inadequately addressed the topic and failed to teach practical assessment skills or cultural considerations (82).

This indicates that the issue is not simply a lack of training, but a fundamental shortfall within the core educational framework of sentinel professions. A systemic reform is therefore needed to place interprofessional education (IPE) at the center of these core curricula. While a recent scoping review identifies comprehensive IPE as essential for strong collaboration, it also highlights a significant lack of well-designed interventions to adequately prepare pre-service professionals (83).

This educational gap is also a symptom of a larger problem: the absence of a clear, consensus-based understanding of a midwife’s specific role in the child safeguarding network. For instance, a Delphi study of Australian nurses and midwives revealed that while they see safeguarding as a core part of their practice, a lack of local or international regulations exists to guide and unify these responsibilities (84). Formally developed practice standards are crucial for guiding curricula, promoting awareness of the midwifery role among other providers, and setting a benchmark for high-quality care.

Therefore, closing the awareness gap identified in our work requires more than just ad-hoc training. It demands the foundational work of establishing and normalizing the expectation that midwives are key players in preventing child abuse. To this end, conducting multi-agency training for students before they begin their careers is particularly important, as it fosters the collaborative skills essential for a sustainable and effective child protection ecosystem.

### 4.4 Limitations

The study’s methodology also has inherent limitations. We chose to measure awareness through self-reported, validated instruments to assess the cognitive and perceptual readiness shaped by different university curricula before students enter their professions. While this approach provides a valuable baseline, we acknowledge a potential gap between a participant’s stated awareness and their actual behavior in complex, real-life safeguarding situations. This discrepancy between self-reported knowledge and practical application is a key limitation. Therefore, future research could build on our findings by using observational or simulation-based methods to explore how this awareness translates into decision-making in practice.

Furthermore, the utilization of convenience sampling within a single public university context limits the immediate generalizability of these results to the broader national or international landscape. It must also be considered that awareness was gauged through self-reported instruments, which may be susceptible to social desirability bias, where students might overstate their perceived competence to align with professional norms. Therefore, while these findings provide a significant indicator of professional preparedness, they should be interpreted with caution regarding their translation into practical application. Finally, although the observed interprofessional differences were statistically significant, the calculated effect sizes (partial η^2^) were relatively small. This suggests that while the academic department is a robust predictor, future research should explore other latent variables—such as clinical placement quality or personal exposure—that may further account for the variance in professional preparedness.

This study’s focus on a single public university in Türkiye presents a limitation. While this approach provides a relevant, in-depth snapshot, the findings may not be directly generalizable to other educational systems or cultural contexts. Future research conducted in varied institutional and national settings would be valuable for developing a broader understanding of these interprofessional dynamics. This limitation does not diminish the significance of the awareness gap identified here; rather, it underscores the need for further research aimed at building a more integrated and effective child protection ecosystem.

## 5 Conclusion

The strength of a sustainable society is measured not only by its economic output but also by its capacity to preserve and nurture human capital across generations. Our research, therefore, moves beyond assessing professional knowledge to identify a critical systemic vulnerability: a significant awareness gap regarding child abuse and neglect (CAN) between sentinel professionals. We argue that this gap constitutes a fundamental breach in social sustainability. This claim is substantiated by our findings, which reveal a nuanced “interprofessional awareness gap” between future midwives and pre-school educators. Although both groups have a commendable knowledge base, the deeper understanding of insidious emotional and economic abuse demonstrated by pre-school educators is not coincidental; it is a direct result of their distinct university education systems.

The source of this disparity is not a lack of practical experience or extra training, but rather the core curricula of the different university programs. This points to a critical conclusion: the educational system itself cultivates professional fragmentation, which in turn creates blind spots in the child protection ecosystem. This issue transcends academic and pedagogical concerns; it poses a direct threat to the long-term resilience and prosperity of our communities. When the health and education sectors operate with different definitions of harm, they cannot form the integrated safety net that vulnerable children require. This systemic failure undermines the development of healthy human capital and perpetuates an intergenerational cycle of trauma—the very antithesis of sustainable development. Ultimately, a society that allows children to be overlooked due to these professional gaps compromises its own future.

To be effective in child safeguarding, professionals must recognize that simple curricular reform is insufficient; a complete paradigm shift in understanding professional responsibility is needed. This shift requires moving beyond isolated, discipline-specific training toward an integrated Interprofessional Education (IPE) model. Such a model must be founded on two cornerstone principles: a shared language of risk and a common understanding of a child’s holistic well-being. A central argument of this paper is that such professional awareness is a necessary precondition for a functional and sustainable social infrastructure. Equipping the next generation of sentinel professionals with a comprehensive, harmonized understanding of their safeguarding duties is therefore not merely a matter of best practice. It is an ethical imperative for building a future that is just, resilient, and truly sustainable for generations to come.

### 5.1 Recommendations

This research indicates the need for a fundamental re-thinking of how we train child protection professionals, moving beyond merely gradual changes. A truly sustainable child protection system requires dismantling the disjointed educational paths that, as this study shows, create interprofessional awareness gaps. This calls for a systemic commitment from universities and policymakers to establish a new, compulsory model of integrated learning that transcends current disciplinary barriers. The core of this model must be an evidence-based, interprofessional curriculum focused on developing a common risk vocabulary and addressing the subtle but damaging effects of emotional, economic, and developmental neglect. Fostering this interprofessional synergy is more than a pedagogical goal; it is a strategic investment in the long-term sustainability of our communities and the well-being of every child. However, implementing such a curriculum requires a shift in pedagogy, not just in content. Experiential and reflective learning methods have proven effective in closing competency gaps in fields like midwifery. For instance, a recent pilot study found that an elective course using e-learning, role-playing, and guided reflection significantly improved midwifery students’ skills in communication, counseling, and therapeutic approaches (85). Critically, that study also revealed that students often recognized their true knowledge gaps only through practical application, having been overconfident beforehand. This highlights that passive, lecture-based learning is insufficient; any new interprofessional curriculum must incorporate active, simulation-based training to move students from theoretical awareness to practical competence.

## Data Availability

The original contributions presented in the study are included in the article/Supplementary material, further inquiries can be directed to the corresponding author/s.
